# A novel CaSR mutation presenting as a severe case of neonatal familial hypocalciuric hypercalcemia

**DOI:** 10.1186/1687-9856-2012-13

**Published:** 2012-05-23

**Authors:** Ksenia N Tonyushkina, Stephen O’Connor, Nancy S Dunbar

**Affiliations:** 1 Baystate Children’s Hospital, Tufts University School of Medicine, 759 Chestnut St. Dept of Pediatrics, Springfield, MA 01199, USA; 2 Baystate Medical Center, Tufts University School of Medicine, 759 Chestnut St. Dept of Radiology, Springfield, MA 01199, USA

**Keywords:** FHH, Neonatal hyperparathyroidism, Hypercalcemia, NHPT, CaSR, Vit D

## Abstract

**Background:**

Familial Hypocalciuric Hypercalcemia (FHH) is a generally benign disorder caused by heterozygous inactivating mutations in the Calcium-Sensing Receptor (CaSR) gene resulting in altered calcium metabolism.

**Objective:**

We report a case of unusually severe neonatal FHH due to a novel CaSR gene mutation that presented with perinatal fractures and moderate hypercalcemia.

**Case overview:**

A female infant was admitted at 2 weeks of age for suspected non-accidental trauma (NAT). Laboratory testing revealed hypercalcemia (3.08 mmol/L), elevated iPTH (20.4 pmol/L) and low urinary calcium clearance (0.0004). Radiographs demonstrated multiple healing metaphyseal and rib fractures and bilateral femoral bowing. The femoral deformity and stage of healing were consistent with prenatal injuries rather than non-accidental trauma (NAT). Treatment was initiated with cholecalciferol, 400 IU/day, and by 6 weeks of age, iPTH levels had decreased into the high-normal range. Follow up radiographs demonstrated marked improvement of bone lesions by 3 months. A CaSR gene mutation study showed heterozygosity for a T>C nucleotide substitution at c.1664 in exon 6, resulting in amino acid change I555T in the extracellular domain consistent with a missense mutation. Her mother does not carry the mutation and the father is unknown. At 18 months of age, the child continues to have relative hyperparathyroidism and moderate hypercalcemia but is otherwise normal.

**Conclusion:**

This neonate with intrauterine fractures and demineralization, moderate hypercalcemia and hyperparathyroidism was found to have a novel inactivating missense mutation of the CaSR not detected in her mother. Resolution of bone lesions and reduction of hyperparathyroidism was likely attributable to the natural evolution of the disorder in infancy as well as the mitigating effect of cholecalciferol treatment.

## Introduction

Familial hypocalciuric hypercalcemia (FHH) is an autosomal dominant disorder characterized by modestly elevated serum calcium (Ca), inappropriately high parathyroid hormone (PTH) levels and low urinary Ca excretion [1]. FHH is caused by inactivating mutations of the gene encoding the Calcium-Sensing Receptor (CaSR), a seven transmembrane G-protein-coupled receptor. The CaSR is expressed primarily in parathyroid chief cells and renal tubular cells and activates an intracellular signaling cascade to maintain serum calcium levels within a narrow range. The gene for the CaSR is located on chromosome 3q13.3–21 and encodes for a 1078 amino acid sequence. Normally, elevated levels of serum calcium activate the CaSR which then inhibits both the secretion of PTH in the parathyroid glands and the reabsorption of Ca^2+^ and Mg^2+^ in the renal tubules. Therefore an inactivating mutation of the CaSR leads to an elevated set point for serum Ca with the consequence of having both inappropriate secretion of PTH and renal reabsorption of Ca^2+^[2].

The clinical impact of these mutations, at least 250 of which have been reported, is highly variable and ranges from totally asymptomatic to fatal. In the neonatal period, homozygous inactivating mutations result in neonatal severe hyperparathyroidism (NSHPT) with severe symptomatic hypercalcemia, undermineralized bones, rib cage deformity, and long-bone and rib fractures [3]. This condition requires aggressive medical management often including emergent parathyroidectomy and can be fatal. Neonatal heterozygous inactivating mutations are usually associated with modest hypercalcemia and hyperparathyroidism with mild or absent bone disease which gradually evolves to a more benign FHH phenotype [4-7]. Lastly, heterozygous FHH can go wholly unrecognized for years until elevated serum calcium is noted [8].

Herein, we present the case of a newborn girl with a novel heterozygous inactivating mutation of the CaSR gene, severe hyperparathyroidism, overt skeletal demineralization, and perinatal fractures who was treated with cholecalciferol to moderate her clinical course.

## Case presentation

**Figure 1:**
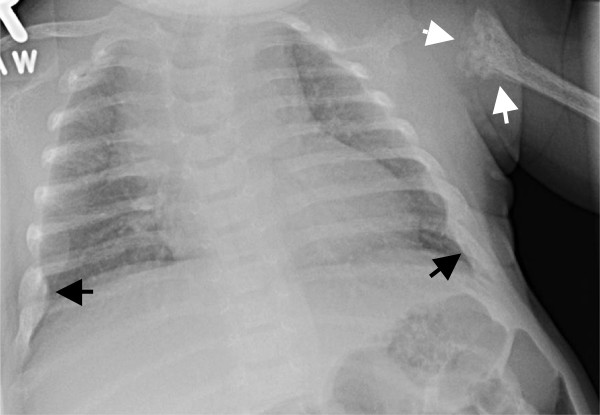
**Chest radiograph at presentation.** There is fragmentation, mixed sclerosis and lucency of the left upper humeral metaphysis and a small amount of subperiosteal new bone formation extending downward along the medial shaft (white arrows). There are expanded healing rib fractures above both CP angles (black arrows).

A full term, healthy infant girl born via a normal spontaneous vaginal delivery presented at 2 weeks of age in a Massachusetts emergency room for evaluation of increased irritability and mild cough. Chest x-ray showed a healing left proximal humeral metaphyseal fracture and bilateral healing ribs fractures (Figure 1) prompting admission for suspicion of NAT.

### Physical exam

She was an alert, well-nourished African-American infant girl whose length was at the 80^th^ percentile, weight was at the 75^th^ percentile and head circumference was at the 75^th^ percentile for age. Exam findings were significant for a prominent forehead and slightly bowed lower extremities. There was full range of motion of extremities without pain.

### Labs

**Figure 2:**
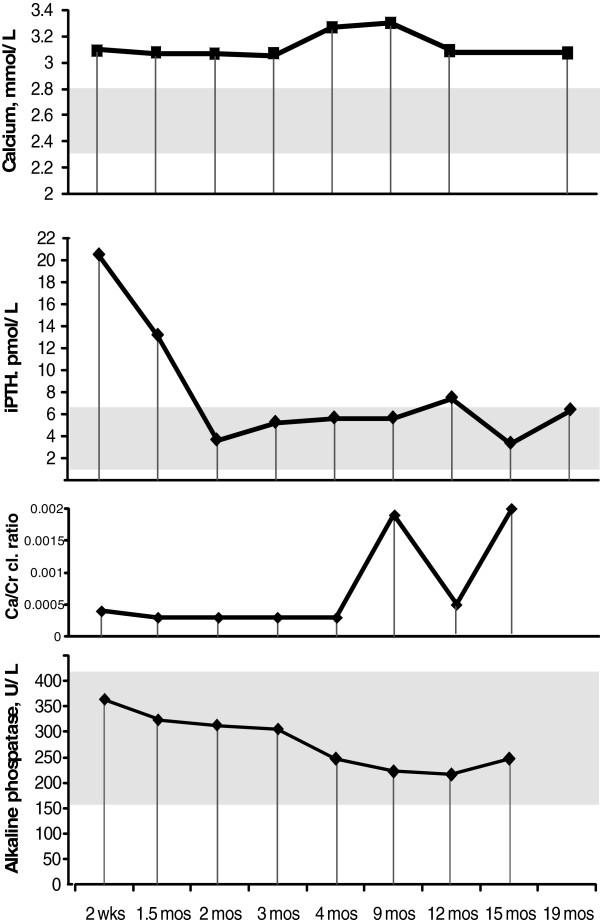
**Serum Calcium, iPTH, Calcium Creatinine Clearance Ratio (CCCR), alkaline phosphatase levels over the course of observation.** Gray zones indicate the reference ranges for each parameter. CCCR reference range not included due to scale; however, values <0.02 consider hypocalciuric.

A markedly elevated intact PTH with mild hypercalcemia and hypermagnesemia were noted on presentation (Table 1). A spot urinary calcium to creatinine clearance ratio was low [9]. After 2 days of hydration with normal saline and low-calcium infant formula (calcium content: <10 mg calcium/100 kCal, Vitamin D 0 IU/oz), serum Ca levels decreased to the high normal range for age (2.8 mmol/L ) (Figure 2).

**Table 1:** Initial biochemical parameters of the infant and mother

**Lab results**	**Initial Lab Results**	
	**Infant, 2 wks**	**Infant’s mother**
Ca {2.3-2.8 mmol/L}; (2.2-2.5 mmol/L)	3.08	2.33
Phos {1.45-2.16 mmol/L}; (0.87-1.45 mmol/L)	1.87	1.42
Mg (0.65-1.0 mmol/L)	1.05	
Cr {25.0-75.0 mol/L}; (41.6-83.3)	25.0	58.3
Alkaline Phosphatase {150–420 U/L}; (30–120 U/L)	364	
Calcium creatinine clearance ratio*	0.0004	0.0005
iPTH (1.0-6.5 pmol/L)	20.4	5.7
Vit D 25(OH) (80–250 nmol/L)	45.18	
Vit D 1,25(OH)2 (42–169 pmol/L)	421.2	

Normal adult ranges are given in parenthesis. Normal ranges for infants up to 24 months are given in curly brackets where applicable.

*CCCR – calcium creatinine clearance ratio = U-calcium/S-calcium/U-creatinine/S-creatinine; calculated with spot urine and serum indices. CCCR <0.02 considered hypocalciuric [9].

### Skeletal survey

**Figure 3:**
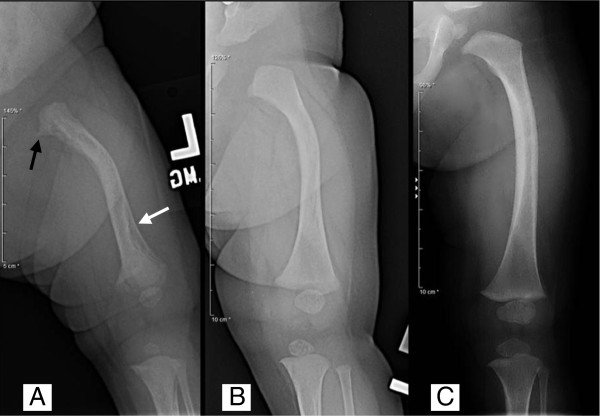
**Changes in radiographic findings of the left femur over the course of observation (age 2 weeks to 1 year). A.** 2 weeks: antero-lateral bowing, widened and irregular metaphyses with flaring and medial beaking (black arrow), and thick, smooth periosteal reaction (white arrow). **B.** 3 months: improved bowing, resolved periosteal reaction and mild residual beaking of the medial upper femoral metaphysis. **C.** 1 year: Further improvement in lateral bowing, complete resolution of metaphyseal deformity.

Bone mineralization was subjectively diminished and there was coarsening of trabecular markings. Repeat CXR with oblique views confirmed multiple healing rib fractures (Figure 1). There was callus formation, flaring, medial beaking and fragmentation of both proximal humeri, the left proximal femur and both distal femurs (Figure 3). There was moderate antero-lateral bowing of both proximal femoral shafts and posterior bowing of the distal femoral shafts with thick confluent subperiosteal new bone formation suggesting advanced stages of healing/remodeling. The femoral bowing deformities and advanced stages of remodeling were consistent with prenatal changes, excluding NAT as a diagnostic consideration. The differential diagnosis included osteogenesis imperfecta and metabolic bone disease.

### Hospital course

The elevated iPTH of 20.4 pmol/L with serum calcium of 2.8 - 3.1 mmol/L led to suspicion of a CaSR mutation. A Sestamibi Tc 99 scan was normal without evidence of adenoma. Lab evaluation of the maternal calcium metabolism was unrevealing (Table 1) and there was no known metabolic bone disease on the maternal side. The infant’s father was unknown. Regular formula was restarted (calcium content 67 mg/100kCal, Vitamin D content 12 IU/oz) and cholecalciferol was given at a daily dose of 400 IU daily to suppress PTH [8]. In sum, her total daily vitamin D intake was approximately 600 IU.

### Follow up course

Over the next 18 months, she continued on cholecalciferol and a regular age-appropriate diet. Her length and weight decreased from the 75^th^ percentile at birth to the 25-50^th^ percentile by 6 months of age but then progressed consistently at the 50^th^ percentile for length and the 25^th^ percentile for weight. The iPTH fell steadily from its peak of 20.4 pmol/L to 4.0 pmol/L by 2 months of age and then settled in the high normal range just under 6 pmol/L. At 12 months of age, the iPTH increased from 5.9 to 7.8 pmol/L so the cholecalciferol dose was increased to 800 IU/day. Calcium levels remained between 3.1 and 3.3 mmol/L with low calcium/creatinine clearance ratios (Figure 2). Follow-up films of the chest and femurs at age 3 months and again at 1 year showed resolution of metaphyseal deformities and periosteal reaction with improving femoral bowing deformities (Figure 3). Renal ultrasound performed at 1 year was normal without evidence of nephrocalcinosis. At her final appointment before being lost to follow-up at age 19 months, her iPTH was 6.6 pmol/L.

### Genetic evaluation

A CaSR gene mutation study (GeneDx DNA diagnostic experts, Gaithersburg, MD) demonstrated heterozygosity for a T>C nucleotide substitution at c.1664 in exon 6, resulting in an amino acid change I555T in the extracellular domain, which replaces an isoleucine with a threonine. This amino acid substitution was predicted by computer modeling performed by GeneDx to be a benign sequence variant although it occurs at a highly conserved region in the CaSR gene [5,10]. The mother’s CaSR mutation testing was normal and the father is unknown.

## Discussion

The severity of the clinical picture in this case including intrauterine fractures was unusual. Classical FHH due to a heterozygous loss of function (LOF) mutation of the CaSR can go unrecognized for years and only rarely is diagnosed in the neonatal period. However, heterozygous forms of FHH with overt neonatal bone disease are described in the literature and are typically defined as NHPT [5-7,10]. Possible causes of the more severe phenotype seen in these cases include the specific type of CaSR gene mutation, paternal versus maternal inheritance, prematurity and maternal vitamin D deficiency [4,8].

### CaSR mutation type

The exact mechanism of how CaSR activation directs intracellular signaling leading to inhibition of PTH secretion and increased reabsorption of calcium in kidneys remains unknown [1]. It is known that the CaSR functions on the cell surface as a homodimer [11]. Inactivation of the CaSR gene can be caused by missense, nonsense and splice –site mutations [10]. The latter two types of mutations lead to truncated CaSR proteins lacking transmembrane or intracellular domains. These proteins cannot dimerize with the wild type CaSR proteins. As a result, in the heterozygous state, calcium sensing will be limited to the 50% structurally normal receptors. CaSR proteins affected by missense mutations can dimerize with the wild type CaSR protein but lead to a malfunctioning heterodimer. In rare instances, the missense mutations may exert a dominant negative effect leaving a minority of wild-type CaSR homodimers functional [4]. Thus, in our case, the presence of a missense mutation may have contributed to the more severe clinical picture but functional studies would be required to further substantiate this possibility. It is important to note that a point mutation in the same codon which also led to an I555T missense change was reported in a patient with FHH and was not present in 94 control patients [10].

### Paternal vs. maternal inheritance

Maternal serum calcium, which is actively transported through the placenta in the third trimester to maintain a maternal-fetal gradient, impacts fetal calcium metabolism. An infant with a maternally inherited mutated allele will have a more benign clinical picture than an infant with a paternally inherited mutated allele because the resulting mild maternal hypercalcemia helps to activate the mutated fetal CaSR [8] and inhibit expression PTH secretion. Conversely, in an infant with a paternally inherited mutated allele, the normocalcemic maternal environment can lead to intrauterine fetal hyperparathyroidism as higher calcium levels would be required to satisfy the mutated fetal CaSR, suppress *PTH* gene expression, and prevent hyperplasia in the fetal parathyroid glands. In our case, the missense mutation of the infant was not found in the mother. Therefore, presumed paternal inheritance or a de novo mutation may have contributed to the unusually severe presentation.

### Prematurity

Prematurity can modify the expected phenotype of neonatal hyperparathyroidism (NHPT) to one more similar to NSHPT. Such a case has been reported previously of an infant born at 27 weeks gestation with moderate hypercalcemia and severe bone disease who was found to have an inactivating mutation (R220W) of CaSR. The infant received serial pamidronate infusions and two parathyroidectomies in an attempt to control the hypercalcemia, but ultimately succumbed to respiratory disease [12]. Because the fetal skeleton depends heavily on maternal calcium and phosphorus transfer during the third trimester, prematurity predisposes an infant to deficient bone mineral accrual. An inactivated CaSR in this setting could lead to further demineralization of an already weakened skeleton and potentially lead to more severe skeletal findings. In addition, the CaSR is expressed in the placenta. Mice studies of inactivating CaSR mutations have found decreased transfer of calcium to the fetus. Theoretically, this could lead to an additional stimulus for fetal PTH secretion.

### Maternal vitamin D deficiency

Maternal vitamin D deficiency with a negative calcium balance has been suggested to cause stimulation of fetal parathyroid glands in utero [8]. The 25(OH)D_3_ in the infant obtained soon after presentation was within the normal range. The mother’s level at delivery is unknown but considering the prevalence of vitamin D deficiency in New England [13] and the fact that this child was born in March, it is plausible that some degree of maternal D insufficiency was involved in the pathogenesis of hyperparathyroidism in this patient, although likely to a small degree.

After 2–3 months of cholecalciferol treatment, the skeletal findings resolved and the biochemical parameters more closely corresponded to the classical features seen in FHH. The infant had a combination of mild hypercalcemia, mildly elevated serum magnesium, inappropriately high-normal PTH and low urinary calcium creatinine clearance [1].

Postnatally, the CaSR is not widely expressed in renal tubular cells but its expression increases over the first several months leading to enhanced calcium reabsorption and declining urinary calcium excretion in the normal infant [14]. In our infant, the calcium/creatinine clearance ratio (CCCR) was strikingly low throughout her course and consistently in the range seen in FHH which is <0.02 [9]. Despite resolution of the severe hyperparathyroidism over the infant’s first 3 months, her PTH continued to be in the high normal range despite increasing cholecalciferol dosing. This might indicate a more severe than average course, since persistent elevation of PTH is reported only in 20% of cases of FHH [10]. In addition, CaSR mutations are associated with adenomatous primary hyperparathyroidism [15].

We elected to use cholecalciferol to try to hasten the resolution of the hyperparathyroidism because of the active bone disease. Within the parathyroid glands, local conversion of 25(OH)D_3_ to 1,25(OH)_2_ D_3_ has been suggested as an independent factor modulating CaSR and PTH production [16]. 1,25(OH)_2_ D_3_ is known to regulate *PTH* and *CaSR* gene expression as well as parathyroid cell growth [17]. Vitamin D has been shown to help decrease PTH levels in primary and secondary hyperparathyroidism [18] and has been used in the treatment of clinically significant FHH [8]. Our experience showed that cholecalciferol treatment was safe. Potentially it may have supported the infant in achieving the elevated threshold for calcium-sensing of the mutated CaSR, but its role in moderating symptomatic FHH or altering the natural course of FHH has to be further investigated. Another medical treatment option for cases such as this would be calcimemetics like cinacalcet. This agent activates the CaSR through binding to the seven transmembrane domain of the CaSR and has been shown to improve the sensitivity of the parathyroid glands to calcium and to treat hyperparathyroidism [19]. Since there is significant genetic heterogeneity in FHH, calcimemetics might not render equivalent effects in different types of CaSR mutations. One might consider using a calcimemetic in the future if the persistent hyperparathyroidism and hypercalcemia leads to complications.

## Conclusion

In summary, this report describes a case of neonatal FHH secondary to a heterozygous inactivating missense mutation within the CaSR gene that presented with severe hyperparathyroidism, overt neonatal skeletal demineralization and perinatal fractures. We reviewed the possible causes of the uncommonly severe clinical picture and its resolution. And finally, we discussed the theoretical role of cholecalciferol in supporting the infant with clinically significant neonatal FHH via PTH suppression. This child will require careful follow-up as her PTH level still remains elevated placing her at risk of clinical hyperparathyroidism in the future as well as monitoring for the development of primary HPT which has been reported in cases of FHH.

## Consent

Informed consent was obtained from the patient’s family for publication of this case and any accompanying images.

## Competing interests

There are no potential conflicts of interest with respect to financial or personal relationships.

## Authors’ contributions

KT acquired the clinical information, drafted and revised the manuscript. ND conceived the case report, helped to draft and revised the manuscript. SO helped to revise the manuscript. All authors read and approved the final manuscript.

## References

[B1] Brown EM Clinical lessons from the calcium-sensing receptor Nat Clin Pract Endocrinol Metab 2007 3 122 133 17237839 10.1038/ncpendmet0388

[B2] Pidasheva S D’Souza-Li L Canaff L Cole DE Hendy GN CASRdb: calcium-sensing receptor locus-specific database for mutations causing familial (benign) hypocalciuric hypercalcemia, neonatal severe hyperparathyroidism, and autosomal dominant hypocalcemia Hum Mutat 2004 24 107 111 10.1002/humu.20067 15241791

[B3] Pollak MR Brown EM Chou YH Hebert SC Marx SJ Steinmann B Levi T Seidman CE Seidman JG Mutations in the human Ca(2+)-sensing receptor gene cause familial hypocalciuric hypercalcemia and neonatal severe hyperparathyroidism Cell 1993 75 1297 1303 10.1016/0092-8674(93)90617-Y 7916660

[B4] Brown EM Editorial: mutant extracellular calcium-sensing receptors and severity of disease J Clin Endocrinol Metab 2005 90 1246 1248 15699544 10.1210/jc.2004-2483

[B5] Obermannova B Banghova K Sumnik Z Dvorakova HM Betka J Fencl F Kolouskova S Cinek O Lebl J Unusually severe phenotype of neonatal primary hyperparathyroidism due to a heterozygous inactivating mutation in the CASR gene Eur J Pediatr 2009 168 569 573 10.1007/s00431-008-0794-y 18751724

[B6] Page LA Haddow JE Self-limited neonatal hyperparathyroidism in familial hypocalciuric hypercalcemia J Pediatr 1987 111 261 264 10.1016/S0022-3476(87)80083-5 3612401

[B7] Wilkinson H James J Self limiting neonatal primary hyperparathyroidism associated with familial hypocalciuric hypercalcaemia Arch Dis Child 1993 69 319 321 10.1136/adc.69.3_Spec_No.319 8215575 PMC1029502

[B8] Zajickova K Vrbikova J Canaff L Pawelek PD Goltzman D Hendy GN Identification and functional characterization of a novel mutation in the calcium-sensing receptor gene in familial hypocalciuric hypercalcemia: modulation of clinical severity by vitamin D status J Clin Endocrinol Metab 2007 92 2616 2623 10.1210/jc.2007-0123 17473068

[B9] Christensen SE Nissen PH Vestergaard P Heickendorff L Brixen K Mosekilde L Discriminative power of three indices of renal calcium excretion for the distinction between familial hypocalciuric hypercalcaemia and primary hyperparathyroidism: a follow-up study on methods Clin Endocrinol 2008 69 713 720 10.1111/j.1365-2265.2008.03259.x 18410554

[B10] Nissen PH Christensen SE Heickendorff L Brixen K Mosekilde L Molecular genetic analysis of the calcium sensing receptor gene in patients clinically suspected to have familial hypocalciuric hypercalcemia: phenotypic variation and mutation spectrum in a Danish population J Clin Endocrinol Metab 2007 92 4373 4379 10.1210/jc.2007-0322 17698911

[B11] Bai M Trivedi S Kifor O Quinn SJ Brown EM Intermolecular interactions between dimeric calcium-sensing receptor monomers are important for its normal function Proc Natl Acad Sci U S A 1999 96 2834 2839 10.1073/pnas.96.6.2834 10077597 PMC15855

[B12] Fox L Sadowksy J Pringle KP Kidd A Murdoch J Cole DEC Wiltshire E Neonatal hyperparathyroidism and pamidronate therapy in an extremely premature infant Pediatrics 2007 120 e1350 1353 10.1542/peds.2006-3209 17974727

[B13] Merewood A Mehta SD Grossman X Chen TC Mathieu JS Holick MF Bauchner H Widespread vitamin D deficiency in urban Massachusetts newborns and their mothers Pediatrics 2010 125 640 647 10.1542/peds.2009-2158 20308219

[B14] Chattopadhyay N Baum M Bai M Riccardi D Hebert SC Harris HW Brown EM Ontogeny of the extracellular calcium-sensing receptor in rat kidney Am J Physiol 1996 271 F736 743 8853437 10.1152/ajprenal.1996.271.3.F736

[B15] Starker LF Akerström T Long WD Delgado-Verdugo A Donovan P Udelsman R Lifton RP Carling T Frequent germ-line mutations of the MEN1, CASR, and HRPT2/CDC73 genes in young patients with clinically non-familial primary hyperparathyroidism Horm Cancer 2012 3 1–2 44 51 22187299 10.1007/s12672-011-0100-8 PMC10358143

[B16] Segersten U Correa P Hewison M Hellman P Dralle H Carling T Akerstrom G Westin G 25-hydroxyvitamin D(3)-1alpha-hydroxylase expression in normal and pathological parathyroid glands J Clin Endocrinol Metab 2002 87 2967 2972 10.1210/jc.87.6.2967 12050281

[B17] Ritter CS Brown AJ Direct suppression of Pth gene expression by the vitamin D prohormones doxercalciferol and calcidiol requires the vitamin D receptor J Mol Endocrinol 2011 46 63 66 21169421 10.1677/JME-10-0128

[B18] Grey A Lucas J Horne A Gamble G Davidson JS Reid IR Vitamin D repletion in patients with primary hyperparathyroidism and coexistent vitamin D insufficiency J Clin Endocrinol Metab 2005 90 2122 2126 10.1210/jc.2004-1772 15644400

[B19] Festen-Spanjer B Haring CM Koster JB Mudde AH Correction of hypercalcaemia by cinacalcet in familial hypocalciuric hypercalcaemia Clin Endocrinol (Oxf) 2008 68 324 325 17803689 10.1111/j.1365-2265.2007.03027.x

